# Feasibility and efficacy of a novel audiovisual tool to increase colorectal cancer screening among rural Appalachian Kentucky adults

**DOI:** 10.3389/fpubh.2024.1415607

**Published:** 2024-07-11

**Authors:** Aaron J. Kruse-Diehr, Derek Cegelka, Elizabeth Holtsclaw, Jean S. Edward, Sarah C. Vos, Melissa Karrer, Katie Bathje, Melinda Rogers, Elaine Russell, Jennifer Redmond Knight

**Affiliations:** ^1^University of Kentucky College of Medicine, Lexington, KY, United States; ^2^Center for Implementation, Dissemination and Evidence-Based Research, University of Kentucky Center for Clinical and Translational Science, Lexington, KY, United States; ^3^Markey Cancer Center, Lexington, KY, United States; ^4^Hawaii Pacific University School of Nursing, Honolulu, HI, United States; ^5^American Cancer Society, Atlanta, GA, United States; ^6^University of Kentucky College of Nursing, Lexington, KY, United States; ^7^University of Kentucky College of Public Health, Lexington, KY, United States; ^8^Kentucky CancerLink, Lexington, KY, United States; ^9^Kentucky Cancer Program, Somerset, KY, United States; ^10^Kentucky Cancer Consortium, Lexington, KY, United States

**Keywords:** colorectal cancer, cancer screening, health communication, Appalachia, rural

## Abstract

**Introduction:**

Residents of Appalachian regions in Kentucky experience increased colorectal cancer (CRC) incidence and mortality. While population-based screening methods, such as fecal immunochemical tests (FITs), can reduce many screening barriers, written instructions to complete FIT can be challenging for some individuals. We developed a novel audiovisual tool (“talking card”) to educate and motivate accurate FIT completion and assessed its feasibility, acceptability, and efficacy.

**Materials and methods:**

We collected data on the talking card via: (1) cross-sectional surveys exploring perceptions of images, messaging, and perceived utility; (2) follow-up focus groups centered on feasibility and acceptability; and (3) efficacy testing in community-based FIT distribution events, where we assessed FIT completion rate, number of positive vs. negative screens, demographic characteristics of participants, and primary drivers of FIT completion.

**Results:**

Across the three study phases, 692 individuals participated. Survey respondents positively identified with the card’s sounds and images, found it highly acceptable, and reported high-to-very high self-efficacy and response efficacy for completing FIT, with nearly half noting greater likelihood to complete screening after using the tool. Focus group participants confirmed the acceptability of the individuals featured on the card. Nearly 75% of participants provided a FIT accurately completed it, with most indicating the talking card, either alone or combined with another strategy, helped with completion.

**Discussion:**

To reduce CRC screening disparities among Appalachian Kentuckians, population-based screening using contextually relevant implementation strategies must be used alongside clinic-based education. The talking card represents a novel and promising strategy to promote screening uptake in both clinical and community settings.

## 1 Introduction

**Figure 1:**
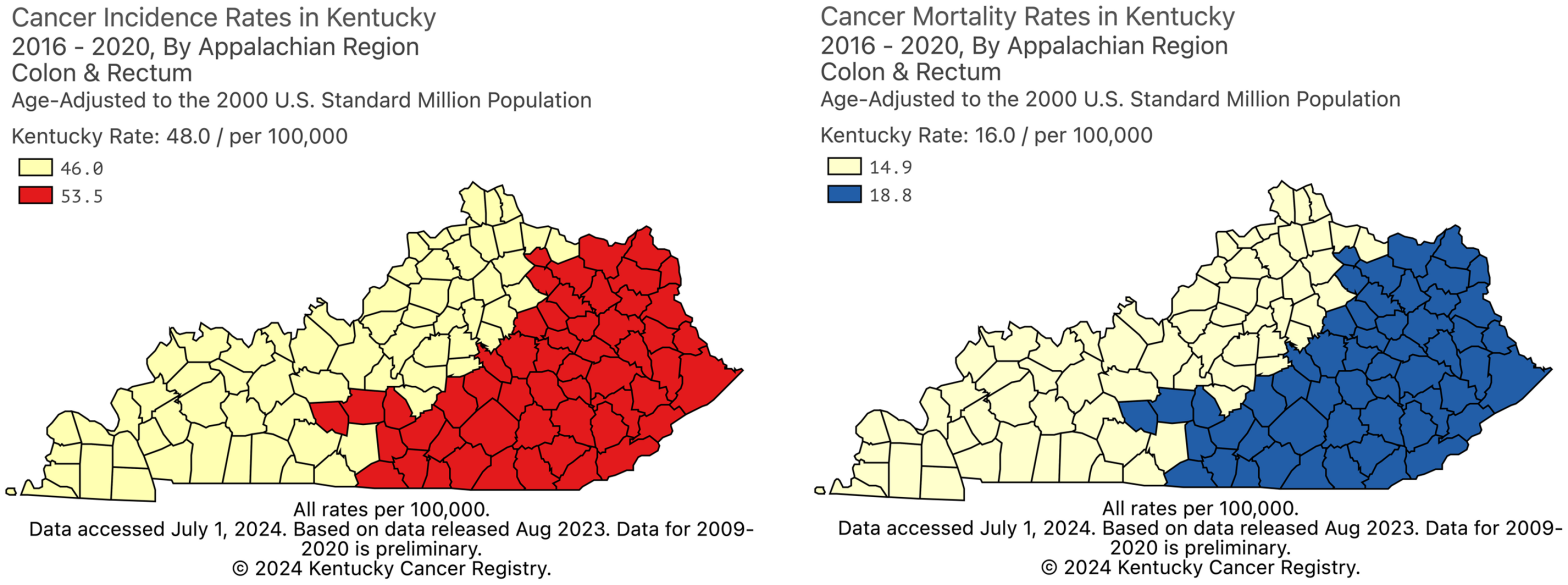
CRC incidence and mortality rates in Kentucky, by Appalachian region.

**Figure 2:**
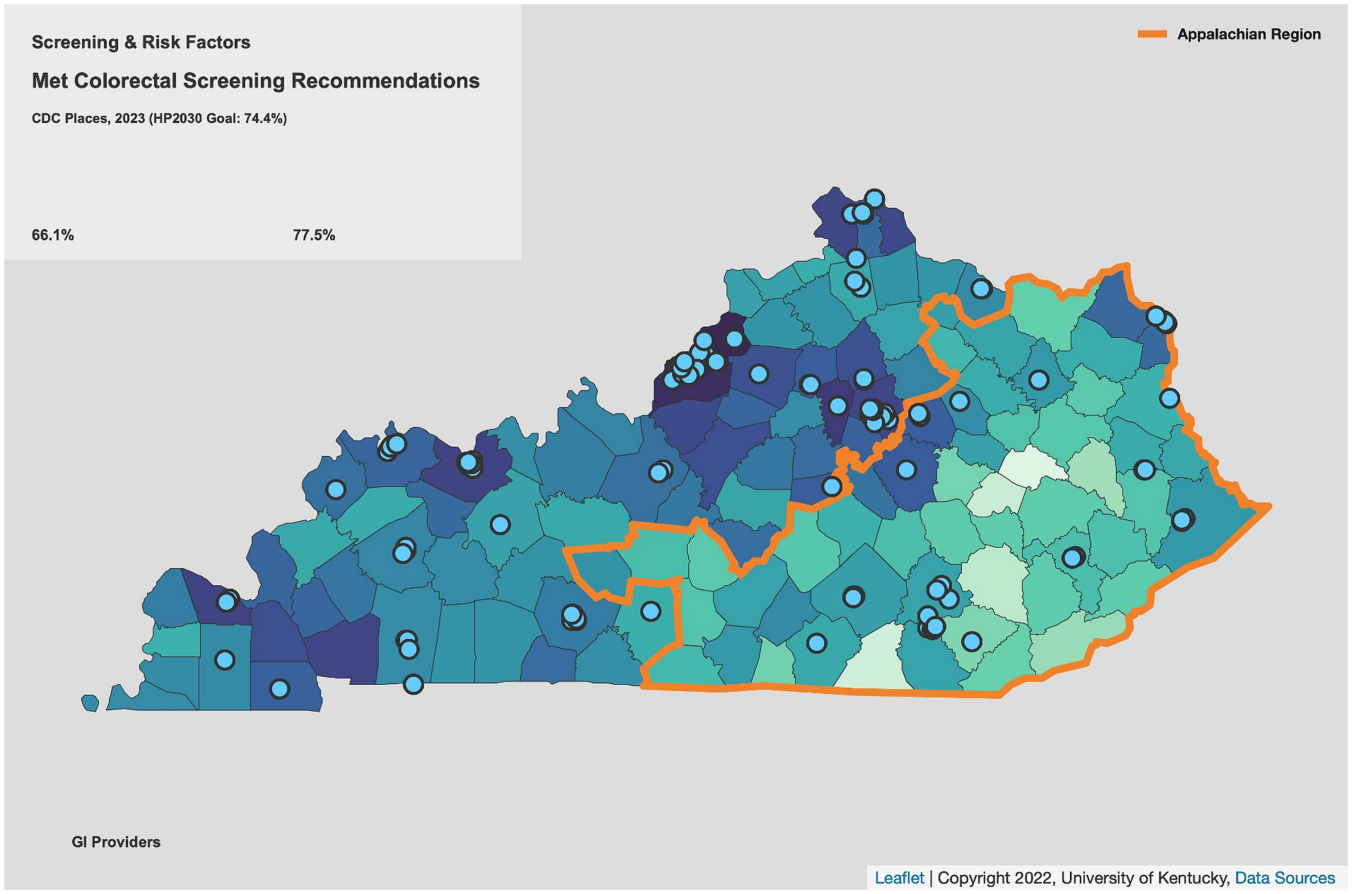
CRC screening rates and distance to GI services in Appalachian Kentucky.

Along with increased colorectal cancer (CRC) incidence (1) and mortality (2) (Figure 1), CRC screening prevalence is lower in rural Appalachian regions of Kentucky than in non-Appalachian regions (3), a disparity partly related to fewer and more geographically dispersed regional specialists available to perform colonoscopy (Figure 2). Individuals living in Appalachian counties tend to earn less money, are more likely to be unemployed, have lower educational attainment, and report poorer health than their non-Appalachian counterparts (4). Additionally, less than a quarter of Appalachian residents hold a bachelor’s degree or higher, a proportion that drops to around 15% for residents living in the most rural parts of Appalachia (4), making health literacy a primary concern for addressing the health needs of Appalachian residents (5). Particularly in rural Kentucky, individuals often live in extremely close-knit communities, and research has shown that Appalachian residents tend to prefer health communication materials reflective of local culture to mass-produced mainstream campaigns (6). Furthermore, addressing patient factors specific to this population–including knowledge of CRC, misperceptions of CRC and screening, fear, and stigma–is critical for increasing CRC screening uptake (7, 8). Methods, materials, images, and communication styles used in screening programs should all reflect local interests, values, and context while simultaneously accounting for varying literacy levels in the intended audience (9).

**Figure 3:**
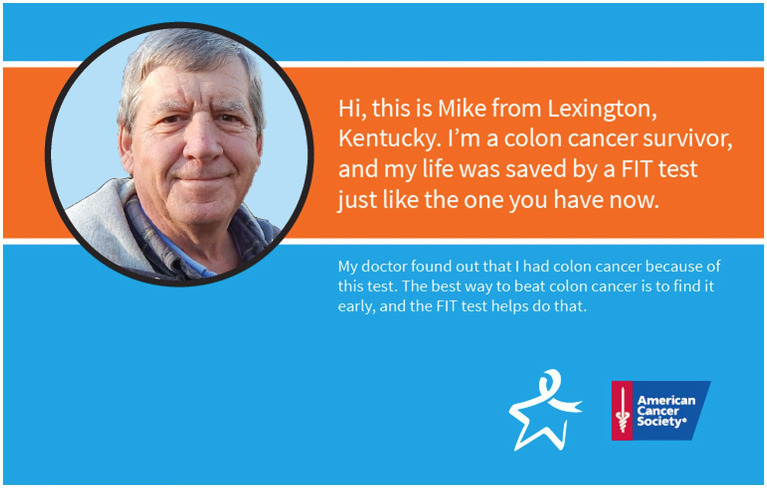
Male-targeted audiovisual tool front cover.

Particularly in rural environments where outpatient services may be limited or geographically dispersed (10), offering a range of evidence-based screening options is critical to increasing overall community screening rates. The use of fecal immunochemical test (FIT) kits as a screening modality has been shown to improve CRC screening by reducing or removing common misperceptions and barriers associated with other screening modalities (e.g., colonoscopy) (11, 12). FIT kits also can be completed in the privacy of one’s home, thereby reducing potential test stigma. Nevertheless, individuals can be confused by the processes required to complete FIT accurately, and instructions included with kits are not always appropriate for low-literacy populations (13). In response to these needs, the Kentucky Cancer Consortium (KCC) (Kentucky’s Comprehensive Cancer Control Coalition) partnered with the American Cancer Society to develop and promote a custom-recordable audio communication tool (“talking card”) intended to help increase CRC screening via FIT. The card provides audio-guided instructions about the importance of CRC screening, the ease of using FIT, and the process for completing a FIT kit. Local CRC survivors from rural Kentucky, one male and one female, are featured on the front of the cards alongside a brief, simple written message about the importance of CRC screening. The inside of the card includes pictorial descriptions of the specific steps needed to complete FIT, as well as audio instructions of those same steps recorded by the individuals on the front of the cards. The talking card size was designed to match the dimensions of the Polymedco OC-Light® FIT mailer, thus allowing them to be used as a potential implementation strategy to increase screening uptake in mailed FIT interventions (Figure 1). The printing cost of the talking card was $3.15 per card, making it an economically feasible strategy to add to a mailed FIT campaign, an evidence-based approach previously proven to be both feasible and cost-effective in eastern Kentucky clinical settings (14) (Figure 3).

For nearly 20 years, one of the focus areas for KCC has been implementing strategies to increase CRC screening by promoting coordination and collaboration among member organizations, which include health care systems. In particular, given the novelty (e.g., simple audiovisual technology that does not require internet connectivity) and contextual focus (e.g., uses images and voices of local individuals with simple audio instructions) of the talking card, KCC wanted to assess both its feasibility and utility before scaling out this strategy to health care systems. Specifically, KCC sought to (a) identify whether the intended population perceived the talking card to be feasible and appropriate and (b) test its efficacy at increasing CRC screening rates. To do this, KCC convened organizational, clinical, and academic partners in a multi-phased effort to explore the feasibility, acceptability and efficacy of the talking cards to increase CRC screening among rural Kentucky residents as part of a mailed FIT campaign.

## 2 Materials and methods

Research on the talking cards have been ongoing since 2018 and have focused both on feasibility and efficacy via three major efforts: (1) cross-sectional surveys exploring perceptions of the cards’ images, messaging, and perceived utility (i.e., feasibility); (2) follow-up focus groups to explore specific characteristics related to the cards’ feasibility and acceptability; and (3) efficacy testing of the talking card in conjunction with community-based FIT distribution events. These efforts were coordinated by KCC in partnership with the University of Kentucky (research assistance), Kentucky Cancer Program (screening/awareness events), the Markey Cancer Center (FIT kits), the Kentucky CancerLink (patient navigation services) and the American Cancer Society (audio supplement cards). All methods, materials, and designs were approved by the University of Kentucky Institutional Review Board or were designated as Not Human Research (NHR) due to being conducted as quality improvement activities within the scope of an organization’s (KCC, Kentucky Cancer Program, Kentucky CancerLink) existing standard operating procedures.

### 2.1 Design, setting and participants

Feasibility and acceptability data for the talking card were collected via a mixed-methods (i.e., QUAN ➔ qual) design consisting of both (1) survey mailings to local screening-eligible patients of three partner family medicine clinics in eastern Kentucky, and (2) two follow-up focus groups with screening-eligible individuals in Appalachian eastern Kentucky. Eligibility criteria for potential participants included being: (1) aged 45–75, (2) a resident of eastern Kentucky, and (3) at average risk for CRC as determined by US Preventive Services Task Force (USPSTF) guidelines (i.e., eligible to use FIT as a CRC screening modality). Surveys were mailed to up to 200 patients randomly selected from each clinic’s list of eligible patients (as determined by their electronic health record system) using a 4-wave survey mailing process (15) (described under “Data Collection” below). Since the focus of the survey was feasibility and because results were intended to be descriptive in nature, there was no power calculation to guide the sample size. Focus group participants were purposively selected with the assistance of community organization partners in eastern Kentucky.

To determine efficacy of the talking cards, outreach partners invited screening-eligible potential participants to local community-clinical linkage events. The events were health-focused, sometimes included a large inflatable colon that participants could “walk through” and were usually part of a larger outreach and awareness event. Events occurred at local hospitals or clinics as well as through community-wide events. Those at risk for colon cancer who participated in the event and showed an interest in the FIT kit had an opportunity to participate. The outreach partners filled out a contact/eligibility form and submitted it to a partner for patient navigation services. The patient navigation partner evaluated the individual’s information and determined eligibility (50–75 years old, screening nonadherent, at average risk). Critically, patient navigators also engaged primary care physicians and insurance companies, when possible, to connect this project with participants’ health care services. To promote consistency across medical records, patient navigators sent either a fax or letter to each participant’s primary care provider with the completed FIT test and attempted to contact their insurance company to provide FIT results.

### 2.2 Data collection

#### 2.2.1 Surveys

Survey mailings featured a 4-wave mailing process (15) in which a packet was sent out to each eligible participant, consisting of six items: (1) a cover letter, signed by a provider at the respective clinic, explaining the study; (2) a brief, simple, pictorial explanation of FIT as a CRC screening modality; (3) a gender-specific version of the talking card; (4) a 3-page survey assessing the talking card; (5) a self-addressed stamped envelope (SASE); and (6) a $2 bill as an incentive. Wave 2 included all items except the $2 incentive, Wave 3 included all items except for the $2 bill and the talking card itself (due to printing cost considerations), and Wave 4 consisted of a postcard reminder. The combined instrument contained both scales created by a health communication expert (SV), as well as previously validated scales. Items assessed self-efficacy (16) and response efficacy regarding FIT, identification with the talking card’s sounds and images, behavioral intentions to get screened for CRC, and perceived acceptability (17) of the talking card.

#### 2.2.2 Focus groups

Follow-up focus groups were facilitated by a qualitative research expert (AK-D) and a community organization partner with extensive experience in community-based cancer education (EH) to contextualize survey findings. Focus group participants were consented, provided photocopies of gender-congruent talking cards and asked to listen as the focus group facilitator opened a talking card and demonstrated its use. The facilitator used a semi-structured interview protocol focused on knowledge of CRC screening and FIT, as well as perceptions of ways in which the talking cards’ messages and images might educate and motivate CRC screening via FIT. Each focus group lasted approximately 1 h. Upon completion, participants completed a survey comprised of three parts: (1) a brief 12-item measure of intervention acceptability, appropriateness, and feasibility (17); (2) a 4-item instrument assessing screening history, recommendation, and perceived barriers; and (3) a demographic component.

#### 2.2.3 Efficacy testing

**Figure 4:**
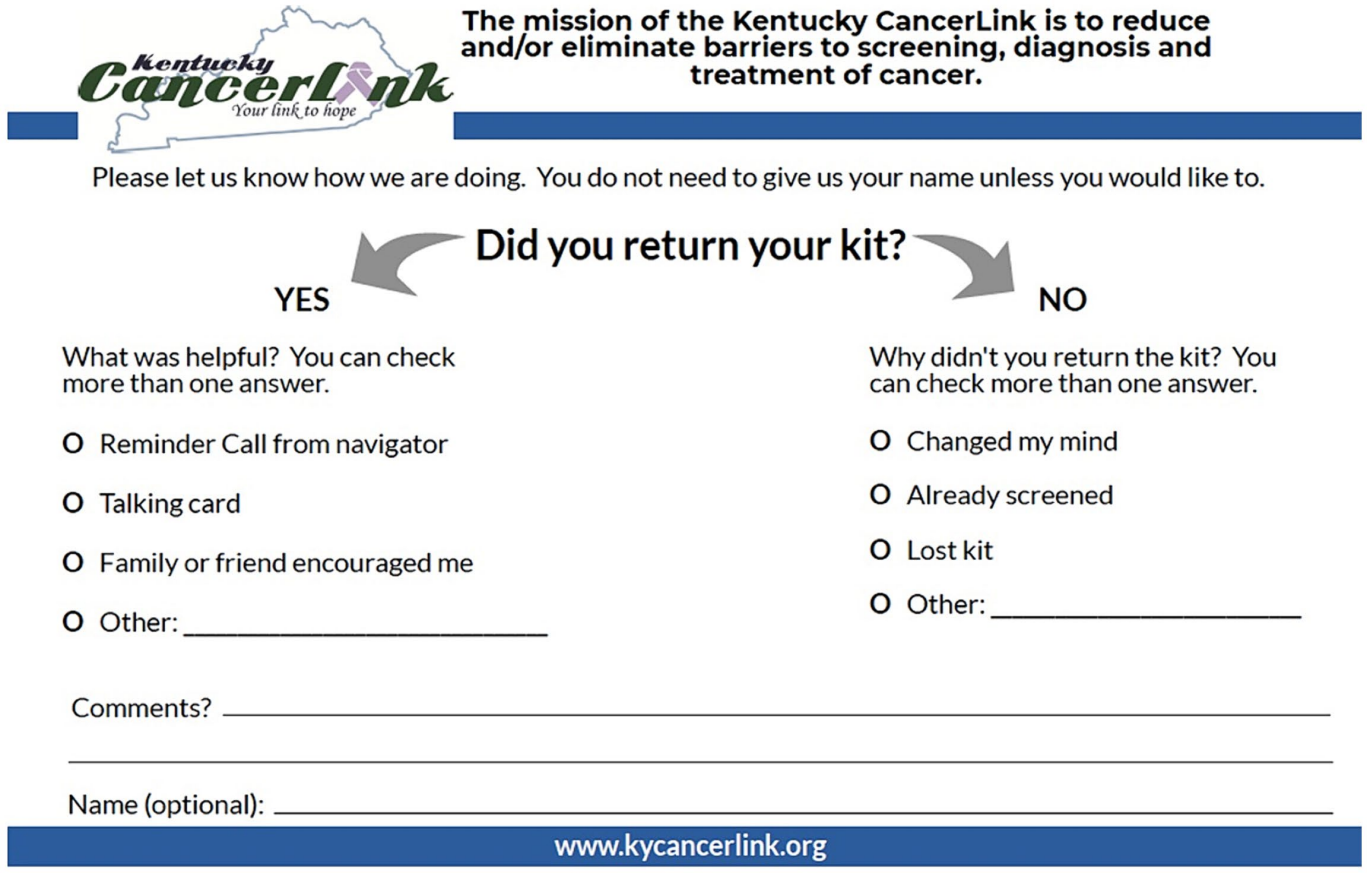
Feedback postcard for community-based FIT distribution campaign.

Finally, statewide community-clinical linkage events were used purposively to collect data on efficacy of the talking cards across three implementation waves. At these events, KCP, Markey Cancer Center and/or Kentucky CancerLink discussed colorectal cancer screening with participants, determined eligibility and had participants fill out eligibility/contact forms which were reviewed by Kentucky CancerLink staff. Those who met eligibility requirements received a mailed FIT kit, talking card, and self-addressed stamped postcard (Figure 4) with an opportunity to provide feedback. Kentucky CancerLink patient navigators contacted participants up to three times and sent a mailed letter to non-responders to assist participants with FIT completion. Upon receipt of FIT, Kentucky CancerLink processed the sample in their CLIA-certified lab; contacted the participant with results; and asked permission to share the results with the patient’s primary care provider, including assisting patients in securing a primary care physician if they did not have one already; and navigating patients with a positive FIT to get a follow-up screening colonoscopy.

### 2.3 Data analysis

Survey data were imputed into an Excel spreadsheet which was uploaded into IBM SPSS Statistics (18) for analysis. Findings from the surveys helped inform focus group questions, which were intended to provide additional context. The two focus groups were audio-taped, transcribed verbatim by a professional transcription service, and spot checked for accuracy by the principal investigator. Transcripts were coded thematically by two members of the research team (AK-D, EH) as per Braun and Clarke (19). Codes related both to broad question topics and were also developed inductively based on conversations that emerged from open conversation within the focus groups and were compiled using a template-based codebook with code operationalizations and exemplars. After individual coding, the investigators met to refine codes and their operationalizations before developing broad themes to describe the focus groups’ primary findings. Although we were unable to apply “member checking” to our themes due to the challenging nature of recruiting our sample, we referenced published literature on CRC screening barriers as well as American Cancer Society community projects to ensure our findings were aligned with prior recent work. Ultimately, no changes were deemed necessary.

Efficacy testing examined the impact of the talking card implementation (i.e., FIT completion rate, number of positive vs. negative screens) as primary outcomes, data on primary drivers of FIT completion from the self-addressed stamped postcard (i.e., any combination of talking card, patient navigation, or family/friend encouragement) as secondary outcomes, and demographic characteristics of participants (i.e., insurance status, race/ethnicity, gender). These data were compiled in an Excel spreadsheet and compared descriptively across 3 waves of implementation.

## 3 Results

### 3.1 Study sample

A total of 692 individuals participated across all three study phases. For the survey mailings, of 353 eligible participants, 67 surveys (19% response rate) were completed and returned. Participants were mainly female (60%), white (98.5%), insured via Medicare (58%), and had a median age of 68 years old (see Table 1). A plurality had a bachelor’s degree or higher (40%) and reported an annual household income of less than $25,000 (25%). A total of 24 individuals participated in the two focus groups. They were also predominantly female (75%), white (91.7%), and between 61 and 70 years old (50%). Most had an educational attainment of associate degree or below (66.6%), a household annual income of between $35,000 and $74,000 (54.2%) and were insured either by an employer plan (54.2%) or Medicare (45.8%). A large majority (87.5%) reported having at least one person they considered their primary medical provider.

**Table 1:** Survey and focus group participant demographics.

	Surveys		Focus Groups	
	*n*	%	*n*	%
*Age*				
45–50	2	3.0	1	4.2
51–55	6	9.0	6	25.0
56–60	10	14.9	1	4.2
61–65	13	19.4	6	25.0
66–70	11	16.4	6	25.0
71–75	23	34.3	3	12.5
76+	1	1.5	1	4.2
No response	1	1.5	0	0
*Gender*				
Female	40	59.7	18	75.0
Male	24	35.8	6	25.0
No response	3	4.5	0	0
*Race/ethnicity^a^*				
White	66	98.5	22	91.7
American Indian or Alaska Native	2	3.0	0	0
Hispanic or Latnix	1	1.5	0	0
Black or African American	0	0	1	4.2
No answer	0	0	1	4.2
*Highest level of education*				
Some high school (did not complete)	6	9.0	2	8.3
High school or GED	14	20.9	6	25.0
Some college (did not complete)	16	23.9	3	12.5
Associate degree	0	0	5	20.8
Bachelor’s degree or higher	27	40.3	8	33.4
No response	3	4.5	0	0
*Total household annual income*				
Less than $25,000	17	25.4	2	8.3
$25,000 to $34,999	6	9.0	1	4.2
$35,000 to $49,999	9	13.4	7	29.2
$50,000 to $74,999	11	16.4	6	25.0
$75,000 to $99,999	11	16.4	2	8.3
$100,000 or more	9	13.4	5	20.9
No response	4	6.0	1	4.2
*Type of medical insurance^a^*				
Medicare	39	58.2	11	45.8
Employer plan (self or spouse’s)	24	35.8	13	54.2
Medicaid	7	10.4	1	4.2
Plan I purchased myself	6	9.0	0	0
Plan through VA	4	6.0	0	0
I do not have medical insurance	2	3.0	1	4.2
Do not know/Not sure	0	0	1	4.2
*Has one or more people considered primary medical care provider*				
Yes	56	83.6	21	87.5
No	9	13.4	2	8.3
No response	2	3.0	1	4.2
*Any type of colorectal cancer screening recommended by medical care provider, ever*				
Yes	51	76.1	21	87.5
No	11	16.4	3	12.5
Do not know/Not sure	5	7.5	0	0
*Stool-based test recommended by medical care provider,past year*				
Yes	15	22.4	–	–
No	49	73.1	–	–
Do not know/Not sure	3	4.5	–	–
*Type of colorectal cancer screening tests taken, past year^a^*				
None	34	50.7	–	–
Colonoscopy or sigmoidoscopy	17	25.4	–	–
Stool blood test like (FIT or Cologuard)	17	25.4	–	–
Other type of colon exam	1	1.5	–	–
No response	2	3.0	–	–
*Type of colorectal cancer screening tests taken, ever*^a^				
Colonoscopy	–	–	22	91.7
Stool blood test (FOBT/FIT or Cologuard)	–	–	6	25.0
Sigmoidoscopy	–	–	3	12.5
CT colonography	–	–	1	4.2
None	–	–	2	8.3
*What reasons for not getting screened for CRC have you heard other people say?^a^*				
Concerned the test is messy	–	–	10	41.7
Worried the test is difficult	–	–	9	37.5
No family history of colorectal cancer	–	–	14	58.3
Belief screening is only for symptoms	–	–	10	41.7
Difficulty finding transportation	–	–	5	20.8
Colonoscopy preparation is unpleasant	–	–	19	79.2
Concerned the test is painful	–	–	11	45.8
Embarrassed to discuss with doctor	–	–	12	50.0
Do not believe they are at risk	–	–	15	62.5
Concerned about costs or insurance	–	–	11	45.8
Difficult to take time off	–	–	20	83.3
None	–	–	1	4.2
*What reasons have kept you from getting screened for CRC?^a^*				
Concerned the test is messy	–	–	0	0
Worried the test is difficult	–	–	1	4.2
No family history of colorectal cancer	–	–	0	0
Belief screening is only for symptoms	–	–	0	0
Difficulty finding transportation	–	–	2	8.3
Colonoscopy preparation is unpleasant	–	–	5	20.8
Concerned the test is painful	–	–	1	4.2
Embarrassed to discuss with doctor	–	–	0	0
Do not believe they are at risk	–	–	1	4.2
Concerned about costs or insurance	–	-	1	4.2
Difficult to take time off	–	–	1	4.2
None	–	–	13	54.2
Other	–	–	2	8.3
*How often do you need help reading written material from the doctor or pharmacy?*				
Never	48	71.6	14	58.3
Rarely	11	16.4	5	20.8
Sometimes	3	4.5	3	12.5
Often	3	4.5	0	0
Always	2	3.0	2	8.3

Surveys: *n* = 66; Focus Groups: *n* = 24. ^a^Percentages do not equal 100% because participants selected all applicable response options.

In efficacy testing, across 3 years of implementation, a total of 601 eligible participants from 73 out of 120 Kentucky counties were identified, 425 of whom (71%) completed a FIT kit. Participants were predominantly female, White, and had some sort of insurance. Only 30 individuals (11%) reported not having any type of insurance.

### 3.2 Feasibility

Survey respondents positively identified with the audiovisual tool’s sounds and images and found it highly acceptable. They also reported high-to-very high self-efficacy (*M* = 3.65, SE = 0.62 on a 4-point scale ranging from *completely disagree* to *completely agree*) and response efficacy (*M* = 3.43, SD = 0.69) for completing FIT after using the audiovisual tool. Nearly half stated they felt better about FIT (46%) and would be more likely to complete screening (48%) after using the tool. While a majority (71.6%) noted never needing help reading written material from the doctor or pharmacy, 12% reported either sometimes, often, or always requiring assistance.

Focus group participants (*n* = 24) stated that CRC screening, in general, is often considered “taboo” in their communities, with one participant stating, “We do not talk about that. You can talk to your kids about [going to the bathroom], but adults…it’s some sort of embarrassing shameful thing.” Other concerns related primarily to colonoscopy, specifically the preparation process and perceptions of discomfort related to the procedure itself. When presented with the talking card, focus group members perceived it to be an improvement over current screening educational materials. One individual commented positively on the audio component, stating that “there [are] probably a lot [of patients] who cannot read or write.” Additionally, the card’s technology was preferred over other approaches such as videos or QR codes due to concerns about spotty internet in rural Kentucky, as well as potential issues with technological literacy. Participants also considered the individuals whose pictures and voices were featured on the cards to be appropriate, noting their Appalachian accents, clear diction, and CRC survivorship; in particular, having a CRC survivor as the face and voice of the card was considered by most to be preferable to a doctor or nurse as the card’s representative. Other participants focused on the uniqueness of the card in general, remarking that they would show it to their family and friends. Furthermore, the simplicity of the card was often cited positively: “[It] just walks you right through it. I mean, step by step. Just bam, bam, bam… And you can listen to it as many times as you want in case you get lost.” Participants universally endorsed the talking card as a strategy to incentivize FIT completion but noted that a primer letter from a physician should be sent first, or people might mistake the mailing as junk mail.

In the summative focus group survey, participants rated the talking card as highly acceptable [average of 4.76 (SD = 0.43) on a 5-point scale, ranging from *strongly disagree* to *strongly agree*], appropriate (*M* = 4.79, SD = 0.36), and feasible (*M* = 4.85, SD = 0.31) to motivate their screening intentions, scores that were reflected in focus group discussion, where every participant noted a high-to-very high level of confidence that they would be able to complete a FIT kit after viewing the talking card. Notably, compared to survey mailings, focus group participants had a higher percentage (20.8%) of participants who reported either sometimes, often, or always requiring assistance reading written materials from the doctor or pharmacy. Table 1 displays participant demographics for both survey mailings and focus groups.

### 3.3 Efficacy

Efficacy testing for the talking card yielded promising findings, with 67% (*n* = 425) of eligible participants accurately completing their FIT kit. Of those completers, 305 (79%) had a negative screen, while 82 (21%) had a positive screen and were navigated to receive follow-up colonoscopy, 42 (51%) of whom completed the procedure, though all patients and their providers were given results of the screen regardless. From those colonoscopies, 24 patients (57% of positive screens) had polyps removed, and one patient was diagnosed with CRC (Table 2). Notably, several patients in the 2nd and 3rd years of the study expressed hesitance to receive a colonoscopy due to rising concerns over COVID-19.

**Table 2:** Wellness event participation demographics.

	Year 1		Year 2		Year 3		Total	
	*n*	%	*n*	%	*n*	%	*N*	%
*Eligible participants*								
Completed FIT	161	87	140	65	124	62	425	67
Did not complete FIT	63	28	75	35	76	38	214	33
*FIT screening results*								
Negative	98	61	113	81	94	76	305	79
Positive	25	20	27	19	30	24	82	21
Follow-up colonoscopy	11	44	17	63	14	47	42	51
Polyps removed	7	64	12	71	5	36	24	57
*Gender^a^*								
Female	140	75	98	70	84	68	–	–
Male	46	25	42	30	40	32	–	–
*Race/ethnicity^a,b^*								
Black/African American	16	9	11	8	14	11	–	–
White	165	89	115	82	99	80	–	–
Hispanic/Latinx	4	2	8	6	9	7	–	–
Other	1	1	6	4	2	2	–	–
*Participant insurance type^a,c^*								
Private	71	38	–	–	62	50	–	–
Medicare	49	26	–	–	26	21	–	–
Medicaid	17	9	–	–	13	10	–	–
VA or government	4	2	–	–	–	–	–	–
Insured	–	–	116	83	–	–	–	–
Uninsured	9	5	19	14	11	9	30	11
No answer	36	19	5	4	12	10		
Counties reached^d^	39	33	49	41	35	29	73	61

*N* = 601. ^a^Gender, race/ethnicity, and insurance data were each collected from all eligible participants in year 1 and from only FIT completers in years 2–3. ^b^Percentages do not equal 100 because participants selected all that apply. ^c^Year 2 data were only collected as insured/uninsured. ^d^Percentage denominator is 120, the total number of Kentucky counties.

A total of 140 participants (33%) who completed a FIT kit returned their postcard with feedback on drivers of screening completion. Most reported that the talking card helped them successfully complete their FIT kit, either alone or in combination with something else, including patient navigation, friend/family support, or other types of support (*n* = 91; 65%). The talking card in combination with follow-up patient navigation was cited by 29% (*n* = 41), and the talking card alone by 14% (*n* = 20), as the primary motivator(s) for FIT completion (Table 3).

**Table 3:** Strategies that helped patients complete FIT.

	Year 1		Year 2		Year 3		Total	
	*n*	%	*n*	%	*n*	%	*N*	%
Post card returned^a^	51	32	58	41	31	25	140	33
Talking card alone	8	16	9	16	3	10	20	14
Talking card + patient navigation	19	37	17	29	5	16	41	29
Talking card alone *or* in combination with any other option^b^	34	67	36	62	21	68	91	65

Percentages do not equal 100 because participants could select more than one response. ^a^Denominator refers to patients who completed FIT. ^b^Other options included patient navigation, friend/family support and a generic “other” response.

## 4 Discussion

The purpose of this study was to explore the feasibility, acceptability and efficacy, of a novel audiovisual tool (“talking card”) among mostly rural Kentucky residents. Stool-based testing, which includes the fecal occult blood test and FIT, is one of two USPSTF recommended (20) screening modalities for CRC screening, along with direct visualization (typically colonoscopy). Screening eligible individuals often cite barriers such as disgust (21), overall cost of the procedure (22), day of procedure requirements for transportation and requesting time off work (23), and fear and embarrassment (24) as common barriers to colonoscopy. The promotion of noninvasive stool-based modalities is particularly important in disparate populations that might be disproportionately impacted by these barriers.

Research has indicated that CRC interventions are most effective when they include multicomponent strategies that address multilevel barriers to screening uptake (25–27). The talking card represents a unique and contextually relevant implementation strategy to increase screenings among residents of Appalachian Kentucky, a region with unique barriers to CRC screening that may contribute to excess CRC mortality (28). Our early, but promising, feasibility and efficacy findings suggest that the talking card would add a strategic layer to improve CRC screening uptake in eastern Kentucky, a region with markedly higher CRC incidence and mortality (1, 2) and lower rates of screening (3). Even for people who may not have adequate health literacy to comprehend medical instructions (5), the talking card can provide clear and relatable audio and pictorial instructions to assist in FIT completion.

Primary care represents an ideal setting to promote CRC screening. The use of *inreach* strategies (29) in the primary care setting, such as patient and provider reminders or use of shared decision making aids, has been shown to be effective at increasing patient screening adherence (30, 31). Inreach focuses on providing cancer screenings to those who already utilize health care services (29) and often includes face-to-face discussions with clinicians to determine CRC screening need based on health history (30, 32). Because of this dedicated one-on-one time with patients, primary care physicians are uniquely positioned to assess patients presenting with CRC-related symptoms and recommend screening colonoscopy (33). In Appalachian Kentucky, these physicians often live in their communities, have developed years of rapport with their patients, and can easily fill this important role. Inreach alone, however, may not be sufficient in every clinical setting, making the use of population-based *outreach* (i.e., mailed FIT) also necessary for diagnosing CRC, particularly among asymptomatic patients or those do not regularly utilize health care services (33).

Ultimately, research suggests using a combination of strategies to augment both inreach and outreach is likely most effective at increasing population-based screening rates (29, 34). Outreach strategies in mailed FIT campaigns include phone calls, follow-up mailers, awareness campaigns, and mass media to reach individuals within the community who are less likely to use medical services consistently (35, 36). In eastern Kentucky, rural clinic personnel have cited time and workload concerns as barriers to promoting CRC screening (28), highlighting the need for outreach strategies in addition to any existing clinic-based inreach. Furthermore, combining multiple outreach strategies has been shown to increase FIT return rates more than using an isolated strategy (27, 37–39), a finding echoed in the present study where nearly two-thirds of participants in the efficacy component noted that the talking card combined with another strategy was most helpful for them in completing their FIT. Although it is not known which specific strategies (or number of strategies) would be most effective at increasing screening uptake in eastern Kentucky, our nascent findings suggest the talking card might be useful as an outreach (e.g., added to a mailed FIT campaign) strategy. Future research should focus on exploring the talking card’s efficacy as an inreach strategy, such as being used by clinicians as a shared decision making tool to promote screening.

### 4.1 Limitations

Although we sought to evaluate the feasibility, acceptability, and efficacy of the talking card comprehensively and across multiple years among Kentucky residents, our findings should nonetheless be interpreted with a few limitations in mind. First, our efficacy testing was conducted in community settings. Screening promotion at community events tends to yield higher uptake than in clinical settings because these events often minimize barriers associated with health care settings (40), including cost. Additionally, individuals who attend health fairs tend to be more health-conscious generally, given that they willingly choose to attend these events, perhaps partly explaining why our FIT screening positivity rate was slightly higher than in other (mostly primary care-based) studies (41, 42). Future studies should consider testing the effectiveness of the talking card in population-based mailed FIT campaigns. Second, whereas screening adherence is critical to reduce late-stage CRC incidence and mortality, modification of risk behaviors is also necessary to prevent CRC; our study did not assess prevalence of risk behaviors, including diet or activity, in our participants, though attendees of the community events were provided educational pamphlets that described health promoting behaviors for preventing CRC. Third, our focus group findings utilized purposive sampling, and it is possible our participants’ views were not representative of those of other individuals living in Appalachian Kentucky, though our study sample largely mirrored the demographic characteristics of Kentuckians as a whole; similarly, survey respondents might differ from nonrespondents in significant ways, including regarding health (and general) literacy. Fourth, the lack of a control group only allows us to make preliminary inferences on the efficacy of the talking card, and future studies should examine its effectiveness in a randomized controlled trial. Fifth, though our talking card represents an inexpensive strategy, costing just over $3 per card to produce, we were unable to collect return-on-investment data for the card; future studies should include a rigorous cost effectiveness analysis, particularly when it is used in primary care settings. Finally, an overwhelming majority of our participants reported having health insurance and at least one person they considered a primary care provider. It is possible, and likely, that both uninsured individuals and those who do not typically access health care services have different perceptions and needs related to CRC screening than the individuals who participated in this study. This possibility nevertheless underscores the importance of conducting population-based CRC screening outreach in Appalachian Kentucky.

## 5 Conclusion

Appalachian Kentucky residents have lower CRC screening rates (3) and subsequently higher CRC mortality (2) than non-Appalachian Kentuckians, necessitating attention to developing and testing strategies that might mitigate barriers and increase screening in this unique population. The talking card represents a novel strategy featuring the voices and images of local Appalachian CRC survivors to motivate and educate about CRC screening. Our findings suggest that Kentucky residents found the talking cards to be feasible, acceptable, and appropriate to promote screening, and our early findings suggest they are effective at increasing FIT return when distributed at community health events. Future research will focus on their utility at increasing screening uptake in clinical settings and in mailed FIT campaigns, particularly in rural, Appalachian regions of Kentucky.

## Data availability statement

The raw data supporting the conclusions of this article will be made available by the authors, without undue reservation.

## Ethics statement

The University of Kentucky Institutional Review Board approved of all phases of this study in accordance with local legislation and institutional requirements. Focus group participants provided written informed consent to participate in this study, and survey recipients provided implied consent via response. Efficacy testing data were determined to be Not Human Research by the University of Kentucky Institutional Review Board because all data were obtained by an outside organization as part of their standard operating procedure, and investigators had no access to personal health information or identifiers when viewing or analyzing data. Written informed consent was obtained from individuals for the publication of any potentially identifiable images or data included in this article.

## Author contributions

AK-D: Conceptualization, Data curation, Formal analysis, Investigation, Methodology, Supervision, Writing – original draft, Writing – review & editing. DC: Writing – original draft, Writing – review & editing. EH: Conceptualization, Formal analysis, Methodology, Writing – original draft, Writing – review & editing. JE: Methodology, Writing – original draft, Writing – review & editing. SV: Methodology, Writing – original draft, Writing – review & editing. MK: Conceptualization, Data curation, Writing – original draft, Writing – review & editing. KB: Conceptualization, Data curation, Writing – original draft, Writing – review & editing. MR: Conceptualization, Data curation, Investigation, Writing – original draft, Writing – review & editing. ER: Conceptualization, Writing – original draft, Writing – review & editing. JK: Conceptualization, Formal analysis, Funding acquisition, Investigation, Methodology, Project administration, Writing – original draft, Writing – review & editing.
